# Topical Curcumin Nanocarriers are Neuroprotective in Eye Disease

**DOI:** 10.1038/s41598-018-29393-8

**Published:** 2018-07-23

**Authors:** Benjamin M. Davis, Milena Pahlitzsch, Li Guo, Shiama Balendra, Parth Shah, Nivedita Ravindran, Giulia Malaguarnera, Claudia Sisa, Ehtesham Shamsher, Hisham Hamze, Abdinasir Noor, Acom Sornsute, Satyanarayana Somavarapu, M. Francesca Cordeiro

**Affiliations:** 1 UCL Institute of Ophthalmology, London, EC1V 9EL United Kingdom; 2 UCL School of Pharmacy, London, WC1N 1AX United Kingdom; 3 Western Eye Hospital, ICORG, Imperial College London, London, NW1 5QH United Kingdom

## Abstract

Curcumin (1,7-bis-(4-hydroxy-3-methoxyphenyl)-1,6-heptadiene-3,5dione) is a polyphenol extracted from turmeric that has long been advocated for the treatment of a variety of conditions including neurodegenerative and inflammatory disorders. Despite this promise, the clinical use of curcumin has been limited by the poor solubility and low bioavailability of this molecule. In this article, we describe a novel nanocarrier formulation comprising Pluronic-F127 stabilised D-α-Tocopherol polyethene glycol 1000 succinate nanoparticles, which were used to successfully solubilize high concentrations (4.3 mg/mL) of curcumin. Characterisation with x-ray diffraction and *in vitro* release assays localise curcumin to the nanocarrier interior, with each particle measuring <20 nm diameter. Curcumin-loaded nanocarriers (CN) were found to significantly protect against cobalt chloride induced hypoxia and glutamate induced toxicity *in vitro*, with CN treatment significantly increasing R28 cell viability. Using established glaucoma-related *in vivo* models of ocular hypertension (OHT) and partial optic nerve transection (pONT), topical application of CN twice-daily for three weeks significantly reduced retinal ganglion cell loss compared to controls. Collectively, these results suggest that our novel topical CN formulation has potential as an effective neuroprotective therapy in glaucoma and other eye diseases with neuronal pathology.

## Introduction

Glaucoma describes a distinctive group of progressive optic neuropathies affecting over 60 million people worldwide and responsible for 8.4 million cases of irreversible blindness^1^. Although several mechanisms have been proposed, glaucoma principally involves the loss of retinal ganglion cells (RGCs)^2^. Elevated intraocular pressure (IOP) today presents the only clinically modifiable risk factor for glaucoma progression^3^, however, many patients continue to lose visual field despite well controlled IOP^4^. Due to the limited effectiveness and indirect nature of IOP modulating therapy, the development of novel therapeutic approaches for the treatment of glaucoma independent of IOP modulation is now sought^5^.

RGC apoptosis has been identified as an early event in glaucomatous degeneration and the inhibition of this process has been advocated as a therapeutic strategy^5,6^. For example, in addition to the well-established IOP modulatory effects of brimonidine^7^, this third generation α_2_ adrenergic agonist is also reported to possess an additional neuroprotective activity in rodent models of glaucoma^8^. Although the mechanism of action remains to be fully described, there is emerging clinical evidence to suggest that topical brimonidine therapy exhibits an RGC preserving activity over and above IOP modulating effects in patients with primary open-angle glaucoma^9^.

Curcumin (1,7-bis-(4-hydroxy-3-methoxyphenyl)-1,6-heptadiene-3,5dione) is a polyphenol extracted from turmeric (*Curcuma longa*)^10^ reported to modulate a range of biochemical processes implicated in neurodegenerative disorders^11^. For example, curcumin has been found to attenuate pathways implicated in the pathogenesis of the most common ophthalmic disorders^12–19^, including: mitochondrial-mediated oxidative stress^20^, inflammatory responses via PPAR-γ agonist activity^21^, down-regulation of COX-2 and iNOS^22^, downregulation of JAK2-STAT3 mediated astrogliosis^23^, β-amyloid aggregation^24^, and anti-angiogenic activity via modulation of the VEGF/VEGFR/K-ras pathway^25^. Supplementing rodent diets with 0.01% to 0.25% curcumin has previously been reported to protect RGCs and microvasculature against ischemia/reperfusion injury via inhibition of Nf-κB, STAT3 and MCP-1 overexpression^26^. More recently, intragastric administration of curcumin (10 mg/kg/day) for 6 weeks in a rodent model of ocular hypertension was reported to result in a significant reduction in retinal microglial death^27^. This equates to a typical human dose of 800 mg/day which has previously been associated with adverse effects such as nausea and diarrhoea in addition to an increase in serum alkaline phosphatase and lactate dehydrogenase levels^28^. Despite the therapeutic potential of curcumin in ophthalmology, however, several key challenges have limited the clinical applicability of this agent including its poor water solubility (~11 ng/mL)^29^ and low bioavailability^10,30^. Single oral administration of between 2 g and 12 g of curcumin in humans yields peak serum concentrations of less than 50 ng/mL^31,32^ and repeated administration of gram doses (0.4 g to 12 g) yields variable peak plasma concentrations typically less than 1 µg/mL, with no accumulation after repeated daily administration for up to 3 months^28,33,34^. The number of capsules each patient was required to take (up to 24 × 500 mg capsules per day) and mild gastrointestinal side-effects precluded higher oral dosing of curcumin in this form.

For treatment of ophthalmic disorders, topical administration is the preferred delivery route as it permits self-administration and localises dosing to ocular tissues, minimising the risk of side effects associated with systemic absorption. Local dosing also offers a method to overcome the extremely low systemic bioavailability, rapid metabolic degradation and clearance of this agent^35^. For instance, in mice only trace amounts (0.41 µg/g) of curcumin are reported to reach the CNS after systemic administration^36^. As typically less than 3% of topically applied small drugs are reported to reach posterior ocular tissues^37^, the extremely limited water solubility of curcumin has been a challenge for the topical administration of this drug. Methods to enhance the solubility of curcumin include; prior solubilization in an alkaline buffer (typically 0.5 M sodium hydroxide), dissolution in solvents such as DMSO or incorporation into nanocarriers^38–44^. Solvents such as DMSO are commonly used to dissolve poorly soluble drug candidates for preclinical investigation, however, the toxicity of this agent, even at low concentrations, is increasingly recognised^45^. Furthermore, dissolution of curcumin in DMSO or alkaline are unsuitable for *in vitro* and *in vivo* applications without further dilution into physiological buffers, and subsequent dilution of these curcumin solutions into aqueous buffers at physiological pH, are frequently unstable and result in rapid precipitation^46^.

To overcome these limitations, nanotechnology approaches can be used to provide hydrophobic environments for poorly soluble drug molecules which persist in a stable aqueous suspension. Further advantages of this approach include nanoparticle-mediated protection of encapsulated drug cargo from hydrolytic or enzymatic degradation and enhanced transport across biological barriers^47^. D-α-tocopherol polyethene glycol 1000 succinate (TPGS) is a non-ionic surfactant that forms stable micelles at concentrations of greater than 0.02% w/w^48^. TPGS is considered a safe pharmaceutic adjuvant by the FDA, which coupled with the observation that this agent can inhibit P-glycoprotein activity^49^ has led to the widespread use of this agent in drug delivery systems^50^. In this study, TPGS was combine with Pluronic F127, a difunctional block copolymer surfactant consisting of a central hydrophobic polyoxypropylene group flanked by hydrophilic polyoxyethylene groups. Pluronic F127 has previously been used to sterically stabilise nanocarriers against aggregation^51^. This, combined with the thermoresponsive properties and high biocompatibility of this polymer, has led to it being widely used for ophthalmic drug delivery applications^52,53^.

The aim of the present study was to develop a curcumin nanocarrier comprising TPGS and Pluronic F127 suitable for use as a topical formulation in the treatment of eye diseases. We describe the development of a novel CN formulation of curcumin solubilizing up to 4.5 mg/mL of curcumin with an encapsulation efficiency exceeding 95%, average particle size <20 nm and good stability for over two months when stored at 25 °C in liquid or lyophilized forms. The neuroprotective potential of this formulation is then assessed in the immortalised R28 retinal precursor cell line^54^ subject to insults that have previously been suggested to model aspects of the retinal environment in glaucoma. These include; cobalt chloride (hypoxia mimetic)^55^ and glutamate induced toxicity^56^. Finally, this formulation was shown to be effective as an eye-drop in reducing RGC loss in two well-established rodent models of optic nerve disease, ocular hypertension (OHT) and partial optic nerve transection (pONT) models^57^.

## Results and Discussion

### Spectroscopic methods can be used to assess curcumin encapsulation efficiency and oxidation state

**Figure 1:**
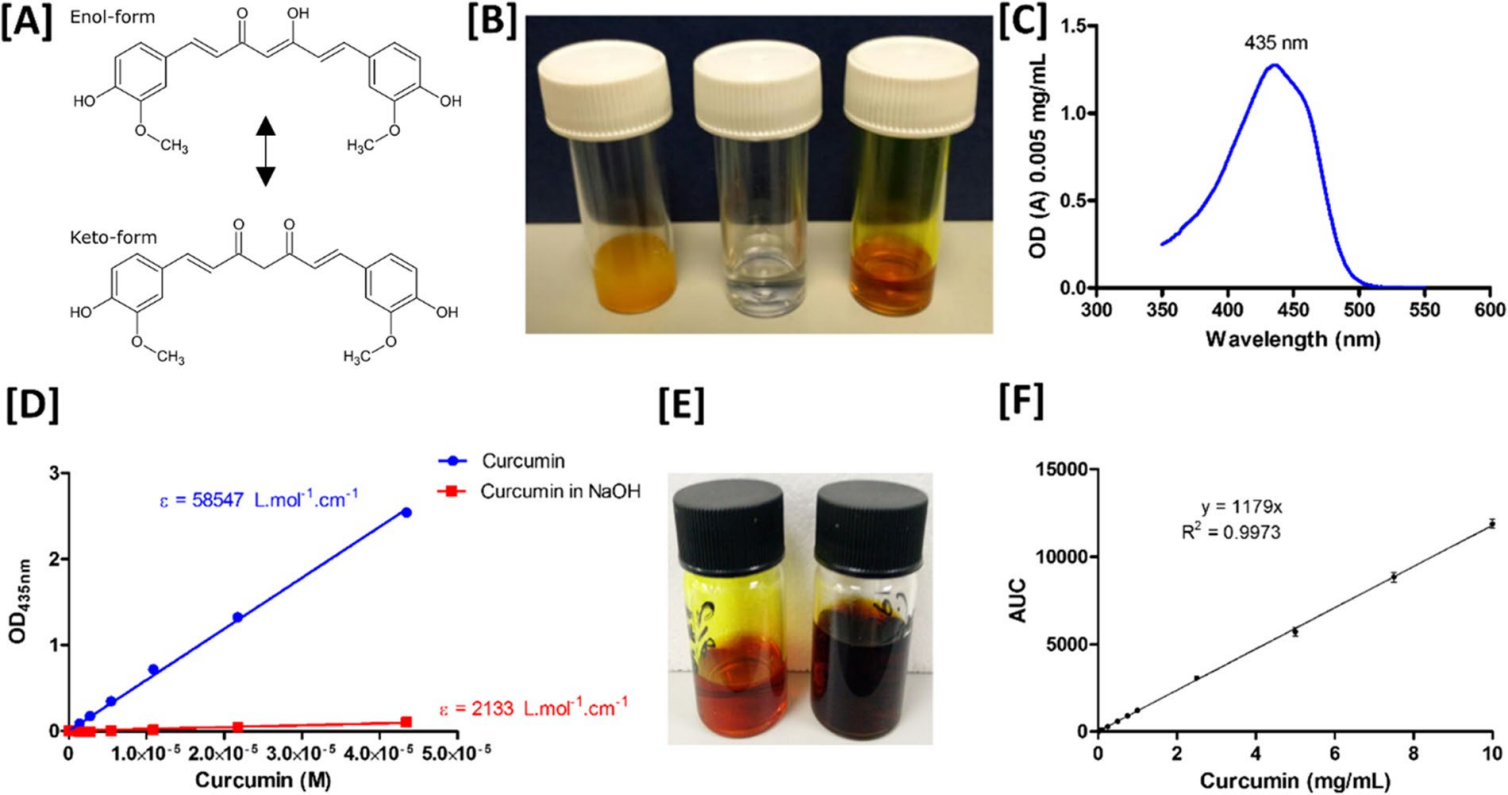
Spectroscopic determination of curcumin content of nanocarrier formulations. (**A**) The keto- and enol- forms of curcumin (1,7-bis-(4-hydroxy-3-methoxyphenyl)-1,6-heptadiene-3,5-dione). (**B**) Suspensions of 4.5 mg/mL of curcumin in *(left)* PBS, *(centre)* PBS after 0.2 µm filtration to remove insoluble material and *(right)* in TPGS/Pluronic F127 nanoparticles after 0.2 µm filtration to remove insoluble material. (**C**) On dissolution in DMSO, 22 µM curcumin possesses an absorption peak of 435 nm. (**D**) Determination of the molar extinction coefficient of curcumin in a DMSO solvent (58,547 L.mol^−1^.cm^−1^) and demonstration that this accelerated oxidative degradation of curcumin at low pH^92^ results in a reduction in this molar extinction coefficient (2133 L.mol^−1^.cm^−1^ after 72 h incubation in 1 M sodium hydroxide solution) suggesting that spectroscopic assessment can be used to monitor both the encapsulation of curcumin and its degradation. (**E**) Dissolution of 5 mg/mL curcumin in 1 M sodium hydroxide solution *(right)* resulting in a rapid colour change compared to curcumin loaded nanocarriers *(left)*. (**F**) Standard curve of known concentration of curcumin measured by HPLC.

On dilution in dimethyl sulfoxide (DMSO) curcumin had an absorbance peak at 435 nm (Fig. 1C) and molar extinction coefficient (Fig. 1D,E) of 58547 L.mol^−1^.cm^−1^, comparable to previously reported values^58^. The absorbance of curcumin diluted in DMSO at 435 nm obeyed Beer-Lambert’s law up to 42 µM and TPGS/Pluronic F127 nanocarriers in the absence of curcumin had no measurable absorbance at this wavelength. Spectroscopic assessment was used to determine the encapsulation efficiency (EE%) of curcumin-containing formulations after separation of unencapsulated material by 0.22 µm filtration. Spectroscopic measurements of EE% were confirmed using an established HPLC technique (Fig. 1F) with both techniques showed good agreement (4.31 ± 0.18 mg/mL versus 4.32 ± 0.33 mg/mL respectively).

Spectroscopic determination of curcumin concentration in nanocarriers can also be used to give indication of the extent of curcumin degradation. Curcumin undergoes keto-enol tautomerization (Fig. 1A), existing in the more stable keto form under acidic or neutral conditions and the more water soluble enol-form under alkaline conditions. In common with other molecules that undergo keto-enol tautomerization^59^, the enol form of curcumin is more prone to hydrolytic degradation^60^. Acceleration of curcumin degradation processes by dissolution in an alkaline buffer^58^, gave rise to a dramatically reduced curcumin molar extinction coefficient at 435 nm compared to formulated curcumin (Fig. 1C,D, 2133 L.mol^−1^.cm^−1^ after 72 h incubation in the presence of 1 M sodium hydroxide solution). Furthermore, incubation of CNs in alkaline conditions induced a dramatic colour change from orange to brown (Fig. 1E). Spectroscopic assessment of curcumin concentration after dissolution in sodium hydroxide indicates that the curcumin molar extinction coefficient rapidly diminished, suggesting that this technique can not only be used to assess curcumin entrapment efficiency but also be used to monitor the extent of degradation of curcumin containing formulations.

### TPGS/Pluronic F127 nanocarriers enhance curcumin solubility and stability

Initially, curcumin loaded nanocarriers were prepared by incorporation it into TPGS nanocarriers. TPGS was chosen due to the low critical micelle concentration of this excipient (0.02% w/w)^48^, the endogenous nature and antioxidant properties of the α-tocopherol component^61^ and P-glycoprotein antagonism^49^, which enhances the barrier crossing ability of formulations containing this agent^62^. TPGS is present in existing ophthalmic formulations^62^ and both curcumin and TPGS can be readily solubilized in ethanol, a solvent which is present at concentrations of 0.8% in commercially available eye drop formulations (i.e. Optrex ActiMist 2in1 Eye Spray for Dry Irritated Eyes) so reducing risks associated with residual solvents from the manufacturing process. Furthermore, as the use of TPGS to enhance the bioavailability of orally administered drugs is well documented^60^. This, in combination with recent interest in the use of Pluronic F127 food-research applications^63^ may suggest that the novel curcumin formulation described herein may also be suitable for oral administration.

Formulation of curcumin with TPGS micelles was found to produce nanocarriers with 16 nm diameter as determined by dynamic light scattering (data not shown). Unfortunately, these formulations rapidly aggregated at 25 °C, resulting in the formation of sediment within hours of resuspension which may be indicative of Ostwald ripening processes^64^. Stabilisation of curcumin loaded TPGS nanocarriers was achieved by the addition of the polymeric stabiliser Pluronic F127 (a triblock copolymer of polyoxyethylene and polyoxypropylene), which has previously been used to sterically stabilise nanocarriers against aggregation^51^.

**Figure 2:**
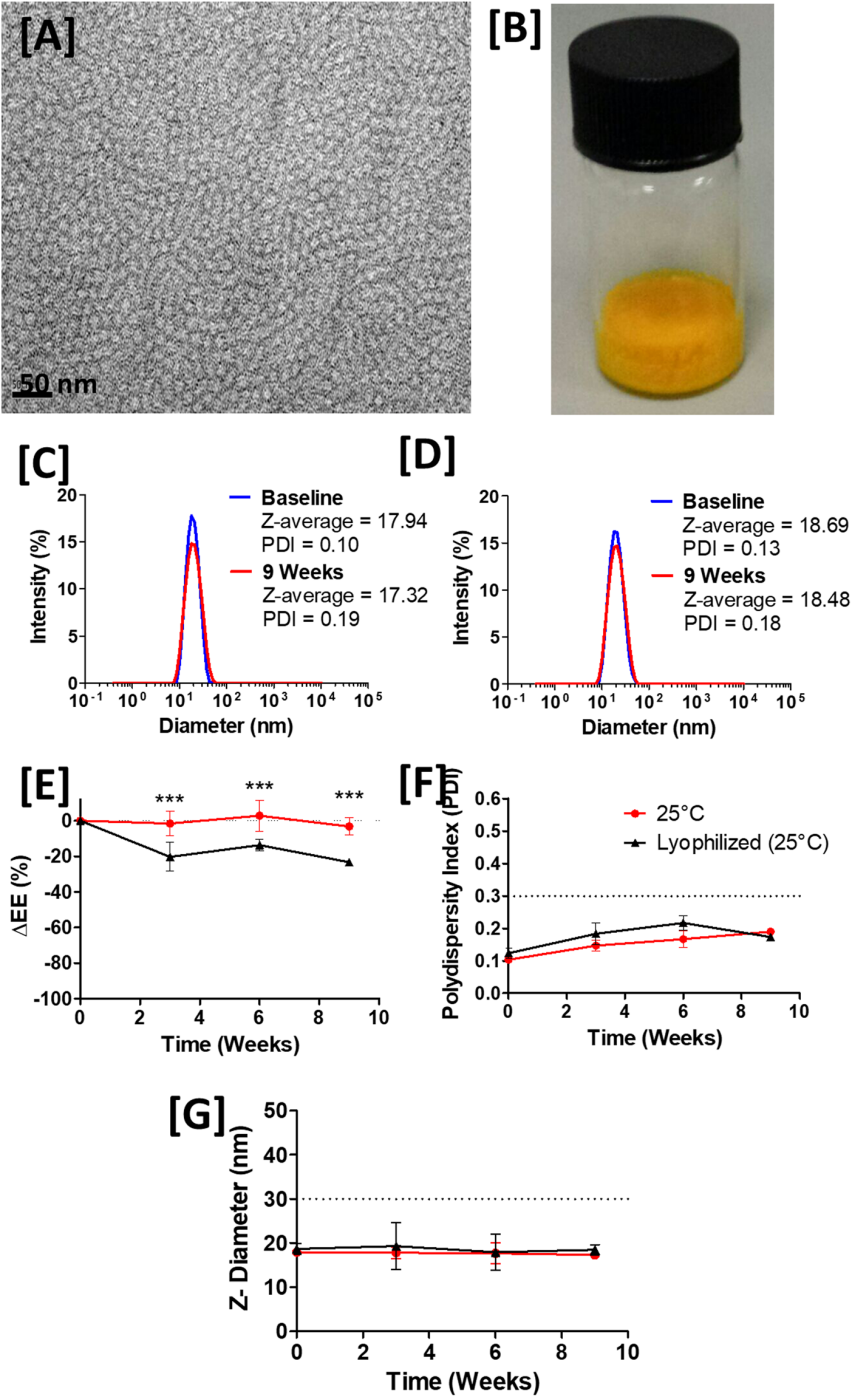
Characterization of curcumin loaded nanoparticles and stability assessment over time. (**A**) Transmission Electron Micrograph of curcumin loaded nanoparticles (CNs) negatively stained with 1% Uranyl acetate. Scale bar = 50 nm. Dynamic light scattering revealed a homogeneous particle population which did not significantly change on storage at (**B**) 25 °C or (**C**) on lyophilization and storage at 25 °C and resuspension after 9 weeks. (**D**) Photograph of 1 mL lyophilized CN in 10 mM HEPES, 50 mg/mL trehalose buffer showing good cake structure. Stability studies illustrating the change in encapsulation efficiency over time when CN were stored at (**E**) 25 °C (solution) versus lyophilized and rehydrated. The average particle size (**F**) and dispersity index (**G**) was recorded in each case. Mean ± 95% CI.

Curcumin-loaded nanocarriers (CN) were prepared according to the methods described, with encapsulation efficiency and average particle size determined (Fig. 2). On resuspension in PBS (pH 7.4) or HEPES trehalose buffer (pH 7.4), nanocarriers were found to encapsulate 96.0% ± 2.0% (4.32 mg/mL) and 94.2% ± 4.1% (4.31 mg/mL) of curcumin respectively. Transmission electron microscopy revealed that nanocarriers were typically 20 nm in diameter and of uniform size (Fig. 2A). These results were confirmed by dynamic light scattering (Fig. 2B,C) which identified a homogeneous particle dispersion with a z-average diameter between 16 and 20 nm suggestive of a micellar formulation.

The encapsulation efficiency and particle size of CN formulations were assessed over time after storage at 25 °C while protecting from light. The CN formulation was found to exhibit excellent stability for 9 weeks at 25 °C, with no reduction in formulation EE% (Fig. 2E), significant change in particle diameter (Fig. 2F) or dispersity (Fig. 2G) over this time. This stability study was repeated using lyophilised CNs prepared in the same buffer before storing at 25 °C while protecting from light. The residual water content calculated at 120 °C was 1.085 ± 0.050%, indicating lyophilized formulations were properly prepared. Formulations were resuspended prior to recording dispersion properties (Fig. 2E,G) which were found to remain constant and similar to those reported for liquid formulations (Table 1). EE% was found to decline by an average of 20% versus baseline at each time point assessed, suggesting this may be a result of the lyophilization or rehydration process.

**Table 1:** Characteristics of curcumin loaded nanocarriers and stability over time (n = 3).

Mean (SD)	Baseline		Three Weeks		Six Weeks		Nine Weeks	
Storage	25 °C	Lyophilized	25 °C	Lyophilized	25 °C	Lyophilized	25 °C	Lyophilized
EE (%)	94.2 (4.1)	101.6 (6.7)	92.6 (1.7)	81.2 (9.7)	97.1 (1.1)	87.8 (1.6)	91.0 (2.2)	78.2 (7.4)
Z-Diameter (nm)	17.9 (0.4)	18.7 (0.5)	17.8 (0.5)	19.3 (2.1)	17.7 (1.0)	17.9 (1.6)	17.3 (0.1)	18.5 (0.5)
PDI	0.002 (0.004)	0.128 (0.029)	0.146 (0.027)	0.183 (0.060)	0.168 (0.046)	0.218 (0.038)	0.188 (0.001)	0.177 (0.0011)

Several groups have previously attempted to prepare curcumin loaded nanoparticle formulations, including; PLGA-nanocarriers^38,39^, solid lipid nanocarriers^40,41^, liposomes^42,43^ and exosomes^44^. Existing nanoparticulate formulations of curcumin possess limited stability (not assessed beyond 72 h in any study cited), only moderate curcumin loading has been achieved (<0.77 mg/mL)^38^ and most protocols would be difficult to translate to the clinic owing to complex, multi-step manufacture protocols requiring organic solvents. The TPGS/Pluronic F127 curcumin formulation described here compares favourably with those in the existing literature.

**Figure 3:**
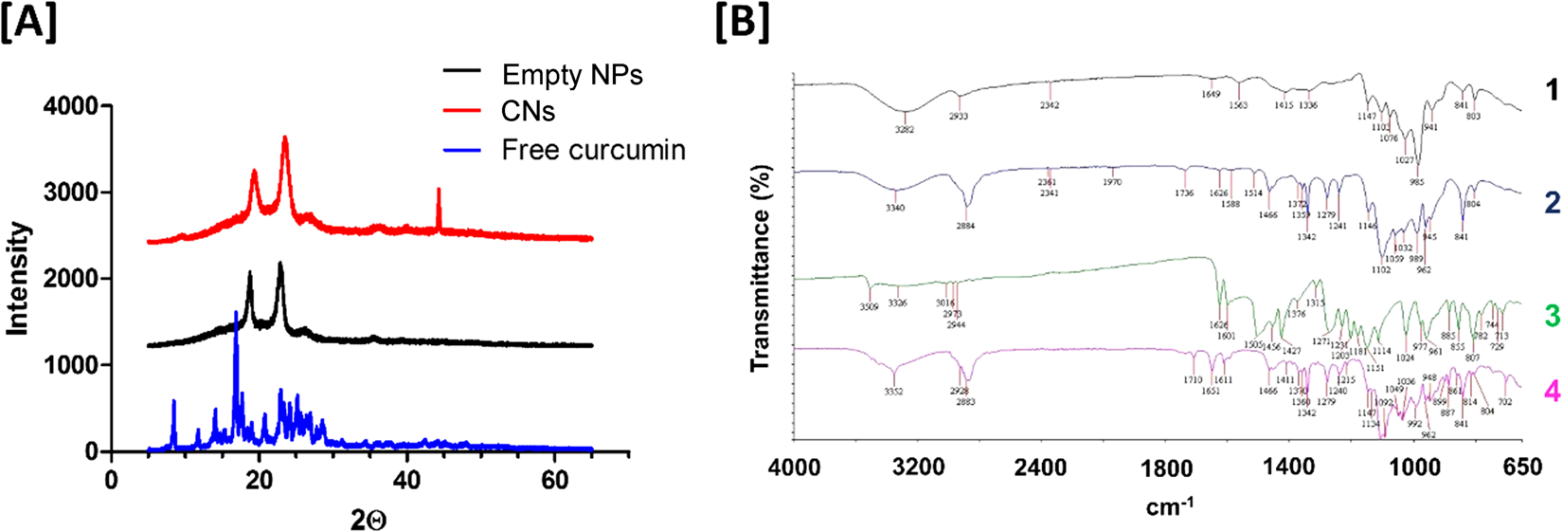
X-ray diffraction and FT-IR characterization of curcumin loaded nanocarrier formulations. (**A**) X-ray diffraction pattern of naïve curcumin (blue), empty nanocarriers (black) and curcumin nanocarriers (red). (**B**) FTIR analysis of (1) trehalose, (2) curcumin loaded nanoparticles, (3) free curcumin and (4) empty nanoparticles.

XRD and FT-IR spectra were acquired to ascertain the nature of curcumin once incorporated into nanocarriers (Fig. 3). The X-ray diffraction patterns of free curcumin exhibited characteristic peaks between 5° and 30°, indicative of a highly crystalline structure^65^. This character was lost on inclusion of curcumin in a nanocarrier formulation, indicating that curcumin has successfully been incorporated into the amorphous nanocarrier structure and is not associated with the particle surface^66^. FT-IR spectra reveal characteristic peaks of free curcumin at 3509 cm^−1^, 1626 cm^−1^, 1601 cm^−1^, 1505 cm^−1^, 1271 cm^−1^, 1024 cm^−1^, 948 cm^−1^ and 713 cm^−1^ which closely match previously reported values^67^. On incorporation into nanocarriers, the characteristic curcumin peak at 3509 cm^−1^ (indicative of the free hydroxyl group) merged with the broad OH peak of the TPGS/Pluronic F127 carrier at 3352 cm^−1^, which may suggest complex formation^68^. Furthermore, characteristics shifts in the aromatic C=C peak (1601 cm^−1^ to 1588 cm^−1^) and the C=O stretching, δ(CCC) and δ(CCO) in plane bending from 1505 cm^−1^ to 1514 cm^−1^ have previously been interpreted as evidence for the successful incorporation of curcumin into a complex^68^.

**Figure 4:**
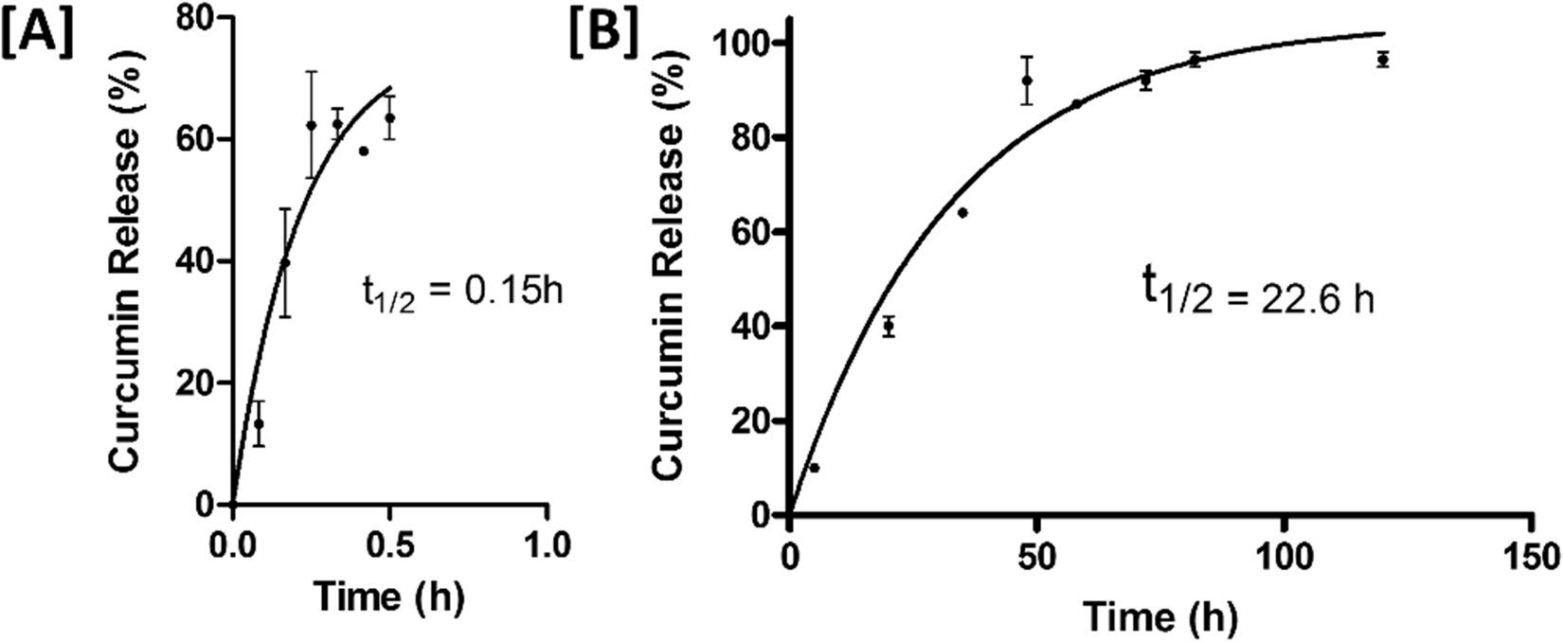
*In vitro* release of curcumin. *In vitro* release of 4.5 mg/mL curcumin from (**A**) 95% ethanol solution or (**B**) curcumin-loaded nanocarriers in PBS at 37 °C (mean ± SE, n = 3). Owing to the poor solubility of curcumin in aqueous buffers, the release of curcumin from ethanoic solutions was limited by the formation of a visible precipitate from the 0.5 h time point. No such aggregation was observed in experiments using nanocarrier curcumin.

Formulation of curcumin into nanocarriers substantially reduced the rate of drug release compared to free drug (t_1/2_ = 22.6 h versus 0.15 h respectively, Fig. 4) at 37 °C, attributed to the slow rate of release of curcumin from nanocarriers. Less than 10% of the drug was liberated after 5 h of incubation, suggesting that there was little burst release from the CN formulation. This observation supports FT-IR and XRD observations that curcumin is not merely associated with the nanocarrier surface but is localised within the hydrophobic interior in an amorphous or disordered crystalline phase, in agreement with previous work^41^. Together, these results suggest that the curcumin-loaded nanocarrier formulation described in this study have sustained release capability.

### Curcumin-loaded nanocarriers protect a retinal cell line against glutamate and hypoxia-induced injury

**Figure 5:**
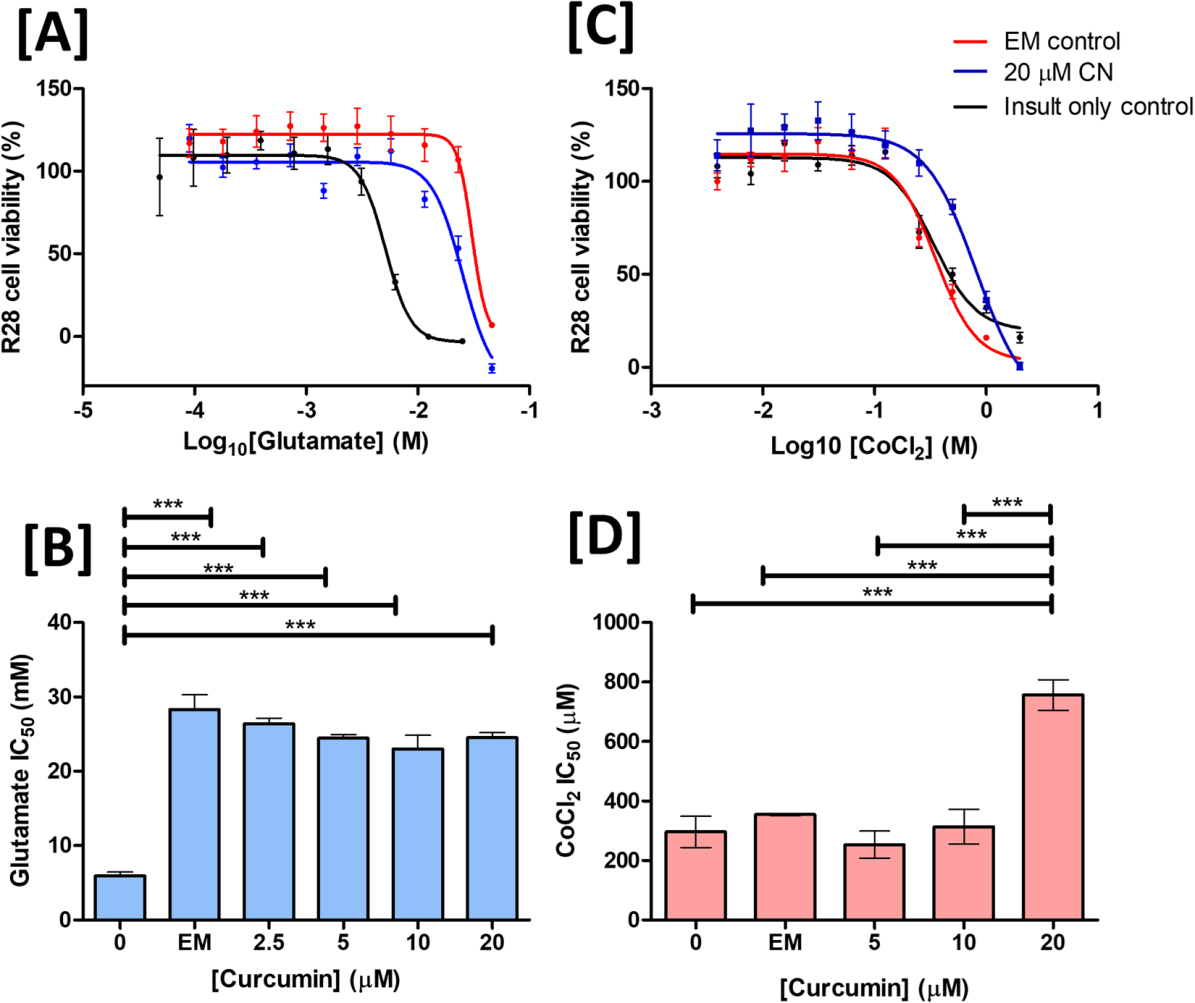
Curcumin nanoparticle treatment is neuroprotective against the hypoxia mimetic cobalt chloride in immortalized retinal cells. Using an alamarBlue cell viability assay, Co-incubation of R28 cells with varying concentration of CNs significantly protected cells against (**A**,**B**) glutamate or (**C**,**D**) cobalt chloride induced insult (one-way ANOVA with Tukey post-test, ***p < 0.001). Empty nanoparticles containing TPGS were found to be neuroprotective against glutamate induced toxicity (**B**) but not cobalt chloride (**D**) induced toxicity.

Glutamate excitotoxicity represents a potential mechanism leading to RGC loss in glaucoma^69,70^. Using an AlamarBlue cell viability assay, co-incubation of immortalised R28 cells with both CNs and empty nanoparticles was found to be significantly protective (one-way ANOVA with Tukey post-test, p < 0.001) against glutamate induced toxicity (Fig. 5A and B, IC_50_ 28.3 ± 3.4 mM versus 5.9 ± 1.2 mM for EM and insult only treated groups respectively, on-way ANOVA with Tukey post-test *p* < 0.001) with no additive effect observed on addition of curcumin to the nanoparticles (24.5 ± 1.2 mM, CN containing 20 µM curcumin). This observation is in agreement with previous studies that suggest α-tocopherol (here present in the form of TPGS) is protective against glutamate induced toxicity and this has been suggested to be a result of the anti-oxidant function of this molecule^71,72^. As TPGS was not also protective against cobalt chloride induced insult, this suggests curcumin and TPGS may have additive therapeutic effects.

Upregulation of hypoxia-related factors such as Hypoxia Inducible Factor 1α (HIF-1α) has been suggested to implicate hypoxia in glaucoma pathology^73,74^. Cobalt chloride (CoCl_2_) is a hypoxia mimetic and inducer of HIF-1α^75^ used as an *in vitro* glaucoma model^76^. The IC_50_ of R28 cells exposed to CoCl_2_ for 24 h (Fig. 5C,D) was found to be significantly increased on concurrent incubation with 20 µM curcumin in the form of CN (296 ± 53 µM vs 757 ± 51 µM respectively, one-way ANOVA with Tukey post-test, *p* < 0.001). Treatment with an equivalent concentration of the nanoparticle in the absence of curcumin had no significant effect (296 ± 53 µM versus 354 ± 8 µM, one-way ANOVA with Tukey post-test, *p* > 0.05) suggesting that the protective effects observed were as a result of curcumin. Concentrations of curcumin <20 µM were not found to be neuroprotective in this model. Curcumin has previously been reported to inhibit HIF-1α in hepatocellular carcinoma cells^77^ and was more recently reported to supresses HIF-1α synthesis in pituitary adenomas^78^. HIF-1α inhibitors have previously been proposed as potential glaucoma treatment worthy of further investigation^79^.

### Topically administered curcumin nanocarrier therapy protects RGCs in rodent models of ocular hypertension and optic nerve injury

Having established the neuroprotective activity of CNs *in vitro* in relation to vehicle only treatments, we next assessed the neuroprotective effects of this formulation on RGC health using an established *in vivo* rodent model of RGC loss. We anticipate that topically applied curcumin loaded nanoparticles will reach the retina via a combination of topical and systemic absorption routes. In support of this hypothesis, Sigurdsson *et al*. reported that their formulation of dexamethasone, which is a similar molecular weight to curcumin (392 versus 368 Da respectively), entered the retina 60% via topical penetration and 40% by systemic absorption route^80^. We anticipate that the well-documented P-gp inhibition activity of tocopherols^49,81^ and curcumin^82^, in conjunction with enhanced corneal penetration activity previously reported for PEGylated-micelle formulations^83^ will enhance curcumin delivery to the retina by the topical absorption route.

**Figure 6:**
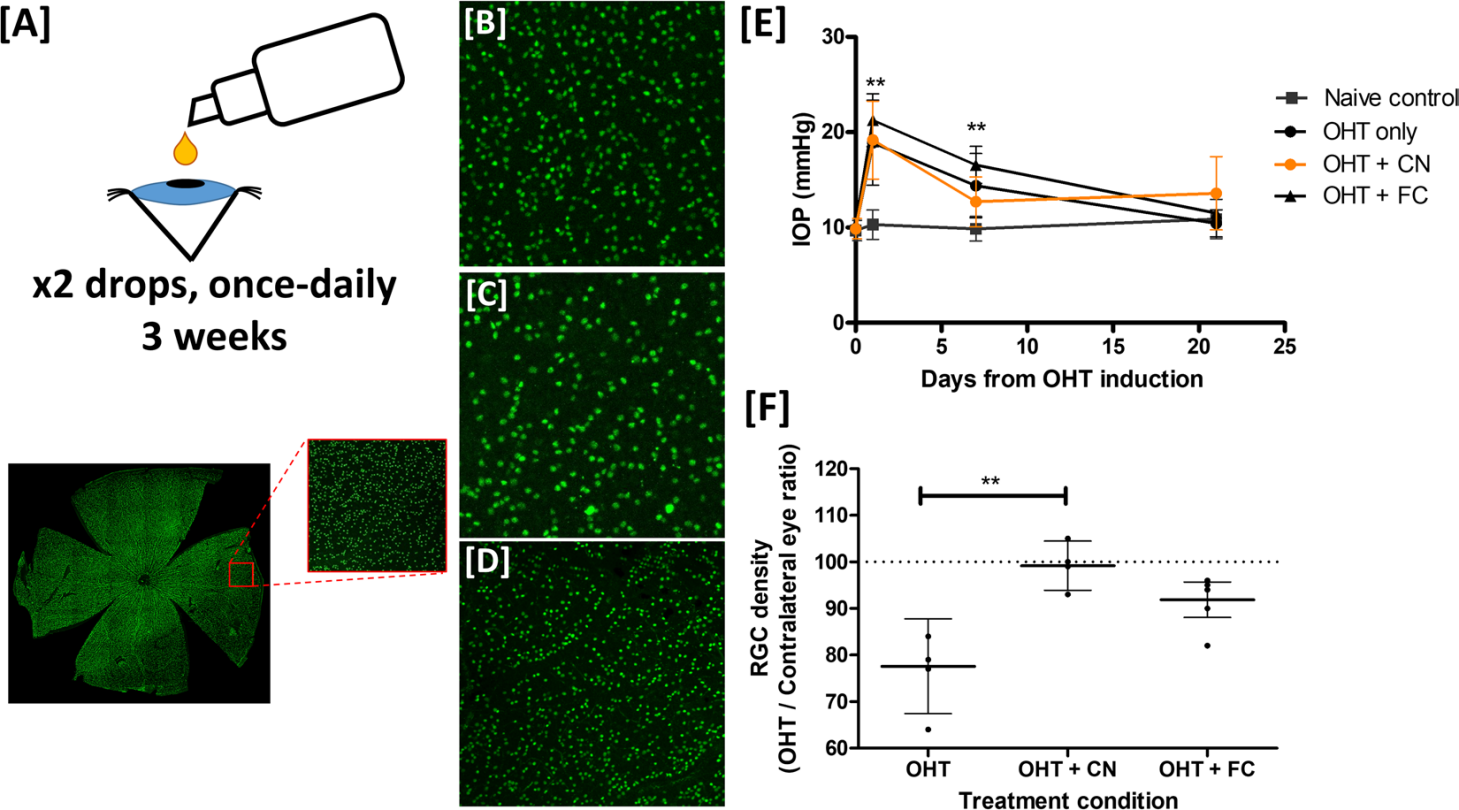
Topical curcumin nanoparticles protect RGC soma *in vivo* against OHT induced cell loss. (**A**) Schematic of *in-vivo* experimental design. OHT rats were randomised to no treatment or once-daily curcumin nanoparticles (CN) or free Curcumin (FC) eye-drops, beginning two days prior to elevated IOP induction. Three-weeks after surgery, animals were sacrificed and retinas flat mounted before labelling with Brn3a. RGC populations were counted as previously described^57,94^. Representative retinal images of comparable Brn3a labelled areas of superior retina are shown in (**B**) Naïve control, (**C**) OHT untreated and (**D**) OHT + CN animals. (**E**) All OHT animals had significantly raised IOP (mean ± SE) versus baseline until 21 days after surgery (Student T-test versus contralateral eyes **p < 0.01). There was no significant difference in IOP between OHT treatment groups at any time point suggesting any neuroprotective activity observed was IOP independent. (**F**) Elevated IOP in OHT only eyes was associated with a significant reduction in RGC density (~23%) in agreement with previous studies^57^; CN but not FC treatment, significantly reduced RGC loss (Kruskal-Wallis test with Dunns post test, **p < 0.01).

Optimum time points post model induction (maximal RGC loss in shortest time after induction) were chosen based on our previous work characterising the natural history of the OHT and pONT models where multiple time points were assessed after model induction^57^. We recently reported that administration of TPGS containing micelles did not themselves have a neuroprotective effect *in vivo*^81^, which in conjunction with our *in vitro* observations, suggest that any neuroprotective efficacy observed was a result of curcumin treatment. Rats received topical CNs according to the dosing regimen illustrated in Fig. 6A. Briefly, two days prior to OHT model induction, rodents began receiving two drops (35 µL each) of CNs dosed five minutes apart per day for three weeks from the date of model induction. Topical administration of CNs was found to be well-tolerated by rats with no signs of ocular irritation or inflammation reported in naive eyes monitored by a qualified ophthalmologist. The IOP profile of rodent’s after IOP elevation by injection of hypertonic saline into two episcleral veins (Fig. 6B) indicates that IOP remained significantly elevated for at least 7 days after model induction versus naive eyes. No significant difference in IOP profile between CN and OHT only groups was observed, suggesting that any neuroprotective effect of curcumin was due to IOP independent processes. RGC health was assessed histologically from whole-retinal mounts using brn3a assessment (Fig. 6C). This approach was chosen as Brn3a is a nuclear restricted and RGC specific transcription factor that exclusively label 97% of the RGC population (excluding photosensitive RGCs)^84^. We have also recently developed an algorithm to accurately and automatically quantify whole RGC populations in rodent models of retinopathy enabling the reliable assessment of RGC health^57^. Using this approach, OHT induction was found to result in a significant reduction in global RGC density of ~23% compared to contralateral eyes, which is comparable to previous studies using this model^57^. CN administration significantly improved the RGC density ratio between OHT eye vs contralateral untreated eyes (Kruskal-Wallis test with Dunns post test, **p < 0.01), whereas administration of un-encapsulated curcumin (FC - free curcumin) solubilised in PBS did not have this effect (Fig. 6D–F).

**Figure 7:**
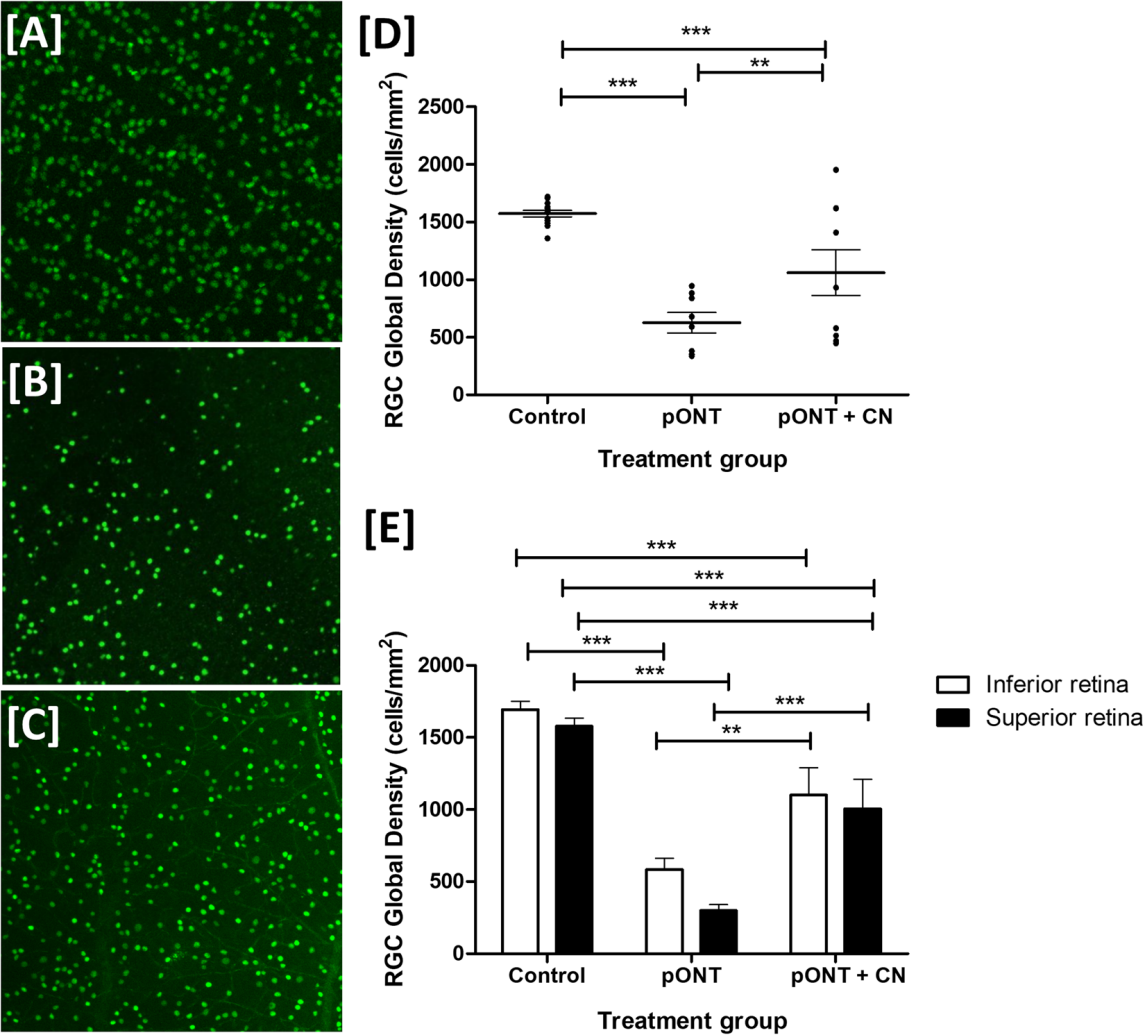
Topical curcumin nanoparticles protect RGC soma against optic nerve injury. Representative Brn3a labelled superior retinal sections taken from similar areas in (**A**) Naïve retina (**B**) pONT retina and (**C**) pONT model retina after daily topical CN treatment for 21 days. (**D**) Whole retinal RGC density measurements indicate that while pONT caused a significant reduction in RGC density, this was reduced by daily administration of CN (one-way ANOVA with Tukey post hoc test, **p < 0.01 and ***p < 0.0001). (**E**) Further segmentation of the each retina into superior and inferior quadrants using the method described previously^57^ demonstrates that topical CN prevent some RGC density loss in both the superior and inferior retina (two-way ANOVA with Tukey post hoc tests ***p < 0.001).

To further investigate the neuroprotective potential of topically applied CNs, whole-retinal brn3a labelled RGC population assessments were made in the pONT model (Fig. 7A). In this model, twice-daily topical administration of CNs was found be significantly protect RGCs (one-way ANOVA, ***p < 0.001). On subdivision of whole retinal mounts into superior and inferior quadrants (Fig. 7B), treatment with CNs was observed to result in preservation of RGC populations in both the superior and inferior quadrants, but this effect was more pronounced in the superior retina (two-way ANOVA, ***p < 0.001), which may imply the protective effects of curcumin therapy exert through anti-apoptotic as well as anti-oxidant mechanisms. Representative regions from the superior quadrant of Brn3a labelled retinal whole-mounts (Fig. 7C–E) illustrate RGC populations were diminished in retina subject pONT (Fig. 7D) versus naive controls (Fig. 7C). Treatment with CNs for three weeks was found to protect RGC soma from pONT induced injury (Fig. 7E). As preservation of RGC soma was observed in both the superior and inferior retinal quadrants, this suggests that curcumin may elicit neuroprotective activity via multiple pathways involving both primary and secondary neurodegeneration processes.

The possibility of TPGS mediated neuroprotection via inhibition of glutamate excitotoxicity is intriguing and may contribute to the neuroprotective effect of our formulation *in vivo*. In support of this hypothesis and our present *in vitro* findings, Nucci *et al*.^85^ previously reported that intraocular administration of a total of 10 µL of 0.5% (w/v) TPGS (equivalent to a total dose of 0.5 mg TPGS) was neuroprotective against ischemia/reperfusion injury in the rat. Previously, we reported that topical administration of TPGS at the same concentration did not have a neuroprotective effect *in vivo*^81^. This discrepancy is likely to the lower concentration reaching the retina compared to invasive application, typically estimated to be ~3% of the topically applied dose^37^. Although our previous work with this model suggests that administration of TPGS only did not appear to have a neuroprotective effect in its own right, a synergism between curcumin and TPGS is extremely likely, if not via the neuroprotective effects of TPGS alone, then perhaps via TPGS mediated modulation of P-gp activity, enhancing curcumin transport across ocular barriers^49,81^.

The neuroprotective effect of curcumin loaded nanocarriers observed in this study may be a result of treatment commencing two days before model induction, suggesting this therapy may be most effective for patients at risk of IOP spikes such as following phacoemulsification surgery^86^ or as a prophylactic to patients identified at high risk of developing glaucoma such as those with ocular hypertension or other glaucoma risk factors^2^. Furthermore, with the development of new techniques such as DARC (detection of apoptotic retinal cells) with the potential to diagnose glaucoma earlier in the disease process^87^, therapies to slow or prevent RGC loss at earlier stages of disease progression will play a greater role in glaucoma management.

In conclusion, this study describes a novel nanocarrier formulation of curcumin in TPGS/Pluronic F127 that increases the solubility of this poorly soluble drug by a factor of almost 400,000. This formulation incorporates 4.3 mg/mL of curcumin with an encapsulation efficiency consistently >90% and excellent stability in liquid or lyophilized forms for at least two months when stored at room temperature, as determined by HPLC and spectroscopic techniques. This formulation was found to be neuroprotective against glutamate and cobalt chloride induced injury in retinal cultures *in vitro* and significantly preserved RGC density in two well-established rodent models of ocular injury. In conclusion, we demonstrate that curcumin loaded nanoparticles have exciting potential for overcoming ocular barriers and may facilitate the translation of curcumin based therapies to the clinic for the treatment of ocular conditions such as glaucoma.

## Methods and Materials

### Preparation of curcumin loaded nanocarriers

Curcumin, D-α-tocopherol polyethene glycol 1000 succinate (TPGS) and Pluronic F127 were obtained at the highest available purity from Sigma-Aldrich (Kent, UK). Curcumin-loaded nanocarriers (CN) were prepared using an adaptation of the thin-film hydration technique described previously^88^. Curcumin, TPGS, and Pluronic F127 were dissolved in ethanol to a concentration of 5 mg/mL, 10 mg/mL and 20 mg/mL respectively; with 10 min of gentle heating and bath ultrasonication to clarity. Solutions were aliquoted in the desired molar ratio (22.55 mM, 12.22 mM 7.94 mM of TPGS, curcumin, and Pluronic F127 respectively) into a round bottom flask, mixing well. The solvent was removed by rotary evaporation (50 mBar, 65 °C, 2 h) using a Rotavapor R210 with a V850 Vacuum controller (Buchi, Switzerland) while protecting from light. After this time, the thin-film was rehydrated (50 °C, 0.5 h) in the desired buffer (distilled water, phosphate buffered saline (pH 7.4) or HEPES trehalose buffer (10 mM HEPES, 50 mg/mL trehalose, pH 7.4). Unencapsulated curcumin was then removed from the formulation by 0.22 µm filtration (33 mm Millex filter, Merck Millipore, USA) as shown in Fig. 1B. Free-curcumin (FC) was prepared according to the same protocol as described above, without the addition of TPGS or Pluronic F127.

### Lyophilisation of curcumin loaded nanocarriers

Lyophilisation of CN formulations in HEPES trehalose buffer was achieved by equilibrating 1 mL aliquots of nanocarriers in 7 mL screw neck squat form glass vials (CamLab, Cambridge UK) at 25 °C before freezing at −60 °C for 2 h at 760 Torr. Primary drying of samples was completed at −38 °C at 200 mTorr for 24 h, followed by a secondary drying phase at 25 °C and 200 mTorr for 2 h. Samples were capped immediately after cessation of secondary drying before storing at 25 °C while protecting from light until required. For stability assessment, samples were rehydrated for 30 minutes by addition of 1 mL of 0.22 µm filtered distilled water with gentle mixing.

The moisture content of formulations was determined using thermogravimetric analysis (TGA). Freeze dry samples were placed in an aluminium pan and analysed by a Discovery TGA (TA instruments, USA). Samples were purged with a flow rate of 25 mL/min nitrogen gas and heated from 30 to 200 °C with 10 °C/min rate. The percent mass loss was calculated by TA Instruments Trios software at 120 °C for water content. Three freeze dry formulations were measured three times for each sample.

### Curcumin loading efficiency

The loading efficiency of CNs was determined spectroscopically and results confirmed using HPLC. Spectroscopic determination of curcumin loading was achieved by diluting in DMSO 1:500 at 435 nm normalised to empty nanocarriers. The concentration of curcumin in each formulation was then determined using the molar extinction coefficient of curcumin (Fig. 1) determined by constructing a standard curve measuring the absorbance of known curcumin concentrations. The encapsulation efficiency of each formulation was calculated using Eq. 1;

1 $$EE \% =100\,.(\frac{{[C]}_{E}}{{[C]}_{S}})$$

where [C]_S_ is the concentration of curcumin originally added to the formulation (typically 4.5 mg/mL) and [C]_E_ is the concentration of curcumin detected spectroscopically within the nanocarriers after 0.22 µm filtration to remove unencapsulated material. Results were confirmed using an adaptation of an established HPLC technique^89^. Briefly, curcumin containing samples were diluted in methanol before 20 µL volumes were injected at 25 °C onto a Phenomenex® Synergi (4 µm Polar—RP 80 A with size of 250 × 4.60 mm) column with an Acetonitrile: 0.1% trifluoroacetic acid 50:50 solvent system at a flow rate of 1 mL/min connected to a Agilent Technology 1260 Infinity HPLC system. Absorbance was recorded at 420 nm and the area under the curcumin elution curve compared to a standard curve of known curcumin concentrations.

### Dynamic light scattering

Particle size was determined using a Malvern Zetasizer. Measurements of particle diameter and polydispersity index were recorded from a minimum of three formulations for each experimental condition or time point after manufacture. Nanocarriers were diluted 1 in 10 in the appropriate buffer prior to recording.

### Transmission electron microscopy

Nanocarrier suspensions were processed using carbon grids to absorb particles from suspension before staining with 1% uranyl acetate for 1 min and drying. Specimens were observed using a Joel-1010 Transition Electron Microscope operated at 100 kV with images acquired using a Gatan Orius digital camera.

### X-ray diffraction and FT-IR

X-ray diffraction graphs of drug alone, empty nanoparticles or CN were prepared from X-ray diffractometer (Rigaku MiniFlex 600) and the 2-thea angle was set from 5° to 65° with an angular increment of 0.05°/second. The measurements were performed at a voltage of 40 kV and 15 mA. The FT-IR spectrum of free curcumin, empty nanoparticles and CN were recorded using a PerkinElmer Spectrum 100 FT-IR spectrometer at 4 cm^−1^ resolution, with 4 scans between 4000 cm^−1^ and 650 cm^−1^.

### Curcumin release assay

*In vitro* curcumin release was assessed using an adaptation of a previously described protocol^41^. Briefly, free curcumin (dissolved in 95% ethanol) or CNs containing 4.5 mg/mL of curcumin was loaded into a 1 mL Spectra-Por Float-A-Lyzer dialysis cassette (Sigma-Aldrich) with 3.5–5 kDa molecular weight cut-off. Samples were dialysed against 200 mL of PBS containing 10% Tween-80 to act as a sink for released curcumin maintained at 37 °C with stirring at 50 rpm. At the specified time points, samples were removed from the mixture and replaced with fresh buffer. The concentration of curcumin was determined as described above. Results from three experimental replicates were fit to a single phase association (Eq. 2);

2 $${\rm{Y}}={\rm{Y}}0+({\rm{Plateau}}-{\rm{Y}}0)\ast (1-\exp (-{\rm{K}}\ast {\rm{x}}))$$

where Y_0_ = zero, Plateau is the maximal release and K is the rate of curcumin release (h^−1^) from which half-life (t_1/2_) was calculated (t_1/2_ = ln2/K).

### Cell culture

R28 cell line (Kerafast, Boston, MA) were cultured in Dulbecco’s modified Eagle’s medium (DMEM; Invitrogen, Paisley, UK) supplemented with 5% foetal bovine serum (Invitrogen, UK), 100 U/ml penicillin, 100 μg/ml of streptomycin and 0.292 mg/mL glutamine (Gibco, UK), 7.5% sterile dH20 and 1.5 mM KCl (Sigma-Aldrich, UK). The medium was changed every other day and cultures were passaged at 90% confluence.

### Cell viability assessment

R28 cells were plated at 4,000 cells/well in 96-well plates for 24 h before treatment with varying concentrations of curcumin (0 to 20 µM) or an equivalent concentration of TPGS/Pluronic F127 only nanocarriers (vehicle control) in conjunction with varying concentrations of cobalt chloride or glutamate insults for a further 24 h. Cell viability was assessed in each case using the Alamarblue (Invitrogen, UK) assay according to manufacturer’s instructions. Briefly, the Alamarblue solution was added to each well-plate and incubated for 4 hours before recording the fluorescence using a Safire plate reader excitation of 530 nm and emission of 590 nm^90^.

### Animals

All animal experiments were performed with procedures approved by the U.K. Home Office and in accordance with the ARVO Statement for the Use of Animals in Ophthalmic and Vision Research. For *in vivo* assessment of experiments: in total 48 Adult male Dark Agouti (DA) rats (Harlan Laboratories, UK) weighing 150 to 200 g were housed in an air-conditioned, 21 °C environment with a 12 h light-dark cycle (140–260 lux), where food and water were available *ad libitum*. 13 animals served as naïve controls which were not subject to further interventions before immunohistochemistry.

### Ocular hypertension (OHT) model

Ocular hypertension was surgically induced in the left eye of 18 DA rats (5 OHT only, 5 OHT + CN, 8 OHT + FC) as described previously^91^. Procedures were conducted under general anaesthesia using a mixture of 37.5% Ketamine (Pfizer Animal Health, Exton, PA), 25% Dormitol (Pfizer Animal Health, Exton, PA) and 37.5% sterile water, at 2 mL/kg administered intraperitoneally. Briefly, 50 µL of hypertonic saline solution (1.8 M) was injected into the two episcleral veins using a syringe pump (50 µL/min; UMP2; World Precision Instruments, Sarasota, FL, USA). A propylene ring with a 1 mm gap cut from the circumference was placed around the equator to prevent injected saline outflow from other aqueous veins. The IOP from both eyes of each rat was measured at regular intervals using a TonoLab tonometer (Tiolat Oy, Helsinki, Finland) under inhalational anaesthesia (0.4% isoflurane in oxygen). Daily administration of topical CNs was performed in 5 DA rats (two 35 µL drops/day 5 min apart at 10 am each day) starting two days prior to model induction and continuing until model termination (21 days post IOP elevation) with 5 rats serving as OHT only controls. An additional 8 rats received free-curcumin (FC) prepared using the same protocol as CN curcumin without the addition of TPGS or Pluronic F127. FC was administered to OHT animals using the same dosing regime as described for CN. Animals were sacrificed three weeks after unilateral IOP elevation and retinas flat-mounted prior to Brn3a immunohistochemistry.

### Partial optic nerve transection (PONT) model

Partial optic nerve transection was conducted in the left eye of 17 DA rats, using a previously described technique^92^. Under general anaesthesia, an incision was made in the superior conjunctiva, and the ON sheath was exposed. A longitudinal slit was next formed in the dura mater to expose the ON and a 0.2-mm cut was made in the dorsal ON, 2 mm behind the eye using an ophthalmic scalpel with steel cutting guard. Damage to major ophthalmic blood vessels was avoided and verified at the completion of surgery by ophthalmoscopy. Daily administration of topical CNs was conducted in 9 DA rats after induction of the pONT model using the same treatment regimen as described previously with the remaining 8 serving as pONT only controls.

### Brn-3a immunohistochemistry and confocal microscopy

Brn-3a labelling of RGCs in retinal whole-mounts was completed as described previously^57^. Briefly, eyes were enucleated upon sacrifice and fixed in 4% paraformaldehyde at 4 °C overnight before dissecting retinal whole mounts. Whole mounts were stained for the RGC specific nuclear-localised transcription factor Brn3a using an anti-mouse mAb (1:500, Merck Millipore, Darmstadt, Germany) and examined under confocal microscopy (LSM 710, Carl Zeiss MicroImaging GmbH, Jena, Germany). Each retinal whole mount was imaged as a tiled z-stack at x10 magnification which was used to generate a single plane maximum projection of the RGC layer in each retina for subsequent analysis. Each whole mount image was manually orientated so that the superior retina was towards the top of the image using *in vivo* cSLO imaging of retinal vasculature as a reference. Retinal image acquisition settings were kept constant for all retinas imaged, allowing comparison of Brn3a expression in each experimental group as previously described^93^. Automated quantification of Brn3a labelled RGCs in retinal whole-mounts was completed as described previously^57^.

### Statistical Analysis

All data were analysed with the Student’s *t*-test, ANOVA or with appropriate post hoc testing using GraphPad Prism 5 (GraphPad Software, Inc., La Jolla, CA, USA) as appropriate. Data were presented as means ± SE and *p* < 0.05 was considered significant. Molecular structures were drawn using ACD/ChemSketch 2015 and all images were taken by the authors (BMD).

### Data availability

The datasets generated during and/or analysed during the current study are available from the corresponding author on reasonable request.
